# No Evidence of QTc Interval Prolongation With Baxdrostat Treatment: Concentration–QTc Modeling Assessment

**DOI:** 10.1002/prp2.70181

**Published:** 2025-10-13

**Authors:** Mikael Sunnåker, Christian Källgren, Joanna Parkinson, Corina Dota, Christer Gottfridsson, David Janzén, Anita Andersson, Glenn Carlson

**Affiliations:** ^1^ Clinical Pharmacology and Quantitative Pharmacology, Clinical Pharmacology and Safety Sciences, BioPharmaceuticals R&D, AstraZeneca Gothenburg Sweden; ^2^ Late‐Stage Development, Cardiovascular, Renal, and Metabolism, BioPharmaceuticals R&D, AstraZeneca Gothenburg Sweden; ^3^ Cardiovascular Safety Centre of Excellence, Global Patient Safety, Oncology R&D, AstraZeneca Gothenburg Sweden; ^4^ Clinical Late Development CVRM AstraZeneca Gaithersburg Maryland USA

**Keywords:** aldosterone inhibitor, baxdrostat, concentration‐QTc modeling, drug safety, hypertension, QT‐interval prolongation

## Abstract

Baxdrostat is a novel, highly potent, selective, competitive inhibitor of human aldosterone synthase currently under development for the treatment of uncontrolled and resistant hypertension and chronic kidney disease. We assessed the risk of QTc‐interval prolongation with baxdrostat using concentration‐QTc (C‐QTc) modeling in healthy adult participants using data from two placebo‐controlled Phase 1 studies: a multiple‐ascending dose (MAD) study of baxdrostat 0.5–5 mg (*N* = 56; NCT05500820) and a Phase 1 four‐way crossover thorough QT/QTc (TQT) study assessing the pharmacokinetics, pharmacodynamics, safety and tolerability of baxdrostat at supratherapeutic doses of 16 and 32 mg (*N* = 28; NCT06194032). In the TQT study, 28 participants were randomized to one of four treatment sequences (each *n* = 7) of baxdrostat 16 mg, baxdrostat 32 mg, placebo and open‐label moxifloxacin 400 mg. Digital electrocardiogram and pharmacokinetic data were collected at baseline and up to 48 h post dose. Dependent and independent variables of the pre‐specified linear mixed‐effect model were placebo‐corrected baseline‐adjusted ΔΔQTcF and baxdrostat plasma concentrations, respectively. Results were consistent between the two C‐QTc modeling analyses. Baxdrostat treatment did not produce QT‐interval prolongation, both at concentrations of interest and geometric mean of the maximum observed plasma concentration. Upper bounds of the two‐sided 90% confidence interval for the ΔΔQTcF mean estimates were < 10 ms. Pharmacokinetic data for the 16 and 32 mg doses in the TQT study were as expected, and both doses were well tolerated. These data illustrate that baxdrostat is not associated with the risk of QT‐interval prolongation at therapeutic and supra‐therapeutic concentrations.

## Graphical abstract

We present concentration‐QTc modeling analyses from two phase 1 studies of the novel, highly selective aldosterone synthase inhibitor, baxdrostat. The assessments presented here confirm the lack of QT‐interval prolongation risk with baxdrostat at therapeutic and supra‐therapeutic concentrations.

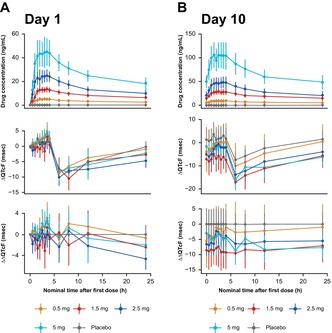

## 1 Introduction

Hypertension is the leading cause of premature death globally [1]. Despite available treatments, many patients have uncontrolled hypertension (uHTN) or resistant hypertension (rHTN), which are associated with poor disease outcomes such as increased risk of cardiovascular events and mortality [2, 3].

Blood pressure (BP) is regulated in large part by the renin‐angiotensin‐aldosterone system [4], of which aldosterone is a key effector [5]. Up to 50% of patients with hypertension may have aldosterone dysregulation, which is characterized by a failure to suppress aldosterone levels in response to high dietary sodium intake, typically in a state of suppressed renin [6, 7]. Aldosterone dysregulation is related directly to BP outcomes including severity of HTN [6, 8], as well as increased risk of adverse cardiovascular and renal disease outcomes independent of BP [9, 10, 11]. The inhibition of aldosterone production is emerging as a potential therapeutic approach for the treatment of hypertension [12, 13].

Baxdrostat is a highly potent and selective aldosterone synthase inhibitor [14], currently being investigated for the treatment of uHTN, rHTN, and chronic kidney disease in Phase 3 trials [15, 16, 17, 18, 19] and primary aldosteronism in a Phase 2 trial [20]. The pharmacokinetics (PK), pharmacodynamics (PD), safety, and tolerability of oral baxdrostat have been evaluated in healthy participants in Phase 1 single‐ and multiple‐ascending dose studies (SAD [baxdrostat 1–360 mg; NCT01995383] and MAD [baxdrostat 0.5–5 mg; NCT05500820]), respectively [14, 21]. In the MAD study, baxdrostat treatment led to a dose‐dependent reduction in plasma aldosterone levels and had no meaningful effect on plasma cortisol levels. Baxdrostat concentrations declined from peak in an apparent biphasic manner, with a terminal half‐life ranging from approximately 26–31 h. The steady state for the PK of baxdrostat, based on the terminal half‐life, is reached in approximately 6 days. The baxdrostat exposure (*C*_max_ and AUC_0‐24h_) was demonstrated to increase proportionally with the dose and, at steady state, an accumulation ratio of ~2 was observed [21].

In a Phase 2, multi‐centre, placebo‐controlled trial in patients with rHTN (BrigHTN; NCT04519658), baxdrostat, compared with placebo, was associated with significant reductions in systolic BP from baseline in all dose groups at Week 12 (statistical significance for baxdrostat 1 mg [−8.1 mm Hg difference versus placebo, *p* = 0.003] and baxdrostat 2 mg [−11.0 mm Hg difference versus placebo, *p* < 0.001]) [12]. There were no baxdrostat‐attributed instances of adrenocortical insufficiency, serious adverse events (SAEs), or deaths [12].

Prolongation of the QT interval can lead to the development of cardiac arrhythmias, such as torsade de pointes (TdP) [22]; therefore, it is essential to characterize the potential of baxdrostat to prolong the QT interval as part of a cardiac safety assessment. For the early evaluation of new non‐antiarrhythmic agents, the International Council for Harmonization (ICH) E14 guidelines recommend a thorough QT/QTc (TQT) study to determine the effects on cardiac repolarisation [22]. Since 2005, the ICH E14 guidelines have been updated through the Q&A process, and Q&A 5.1 (2015) allows, under certain conditions, the use of early‐phase data and concentration‐response modeling as primary analyses to provide TQT substitute data [23].

To date, there has been no evidence of meaningful changes in ECGs including QTc‐interval prolongation during the clinical baxdrostat programme [21, 24, 25]. In order to assess QT prolongation risk, a concentration‐QTc (C‐QTc) analysis was initially conducted using digital ECG (dECG) data collected during the MAD study and included doses up to 5 mg. However, these data were deemed insufficient for QT assessment of baxdrostat given the low doses used. Data from the SAD study with baxdrostat doses up to 360 mg could not be used for the QT assessment due to insufficiencies in the ECG data collection. Therefore, a Phase 1 four‐way crossover TQT study was designed in line with ICH E14 guidelines to evaluate the effect of higher exposures to oral baxdrostat (single baxdrostat 16 and 32 mg [supratherapeutic] doses) in healthy participants, to fully assess the risk of QT‐interval prolongation (NCT06194032). Here, we report the findings of the primary C‐QTc analyses of baxdrostat from the MAD and TQT studies, alongside the full PK, PD, safety, and tolerability results from the TQT study.

## 2 Methods

### 2.1 Baxdrostat 0.5–5.0 mg: Phase 1 MAD Study and C‐QTc Analysis

#### 2.1.1 Study Design

Briefly, this was a Phase 1 randomized, double‐blind study to assess the PK, PD, safety, and tolerability of baxdrostat up to 5 mg in healthy adult participants aged 18–55 years (see Methods S1 for inclusion and exclusion criteria). Complete methods and results have been previously reported [21].

Participants were randomized 3:1 within five cohorts to receive baxdrostat or matching placebo once daily for 10 days: (A) baxdrostat 2.5 mg [*n* = 9] or placebo [*n* = 3] on a low‐salt diet; (B) baxdrostat 5.0 mg [*n* = 9] or placebo [*n* = 3] on a low‐salt diet; (C) baxdrostat 1.5 mg [*n* = 9] or placebo [*n* = 3] on a normal‐salt diet; (D) baxdrostat 2.5 mg [*n* = 6] or placebo [*n* = 2] on a normal‐salt diet; and (E) baxdrostat 0.5 mg [*n* = 9] or placebo [*n* = 3] on a normal‐salt diet. Cohorts A and B were on low‐salt diets to stimulate aldosterone production and evaluate safety in patients who may follow a low‐salt diet (see Methods S1 for a full description of the low‐salt diet). These participants also underwent an adrenocorticotropic hormone (ACTH; Cortrosyn) challenge to increase aldosterone and cortisol levels, for comparison with Cohort D (without Cortrosyn challenge) to evaluate the specificity of baxdrostat for aldosterone synthase.

Blood samples were collected to evaluate PK, and 12‐lead continuous dECG data were collected for a C‐QTc analysis (see Methods S1 for full data collection methods).

#### 2.1.2 Exploratory Analyses

Exploratory graphical analyses (see Methods S1 for pooling methodology and software used) were performed prior to C‐QT modeling to assess the following: (1) the effect of baxdrostat on heart rate (HR); (2) the time delay between baxdrostat concentration and baseline‐adjusted QTc (ΔQTc) and placebo‐corrected ΔQTc (ΔΔQTc) (to determine if the QTc time course was concordant with baxdrostat concentration and exclude hysteresis); (3) the impact of Cortrosyn challenge on the QTc interval; and (4) the linearity of the C‐QTc relationship, corrected for heart rate using the Fridericia formula (QTcF=QT/RR/100013) (to determine the adequacy of using a linear model for describing the C‐QTc relationship).

#### 2.1.3 C‐QTc Analysis

The objective of the C‐QTc analysis was to estimate the placebo‐corrected and baseline‐adjusted QTcF interval (ΔΔQTcF) within the clinically relevant baxdrostat exposure range investigated in the MAD study, using a pre‐specified linear mixed‐effect model with dECG and time‐matched drug concentrations (see Methods S1 for model equations and the software used). A prolonging effect on QTc was excluded if the upper bound of the two‐sided 90% confidence interval (CI) for model‐derived ΔΔQTcF was estimated to be < 10 ms (consistent with ICH E14 guidance) [22, 23] at the highest clinically relevant exposure.

### 2.2 Baxdrostat 16 and 32 mg: Phase 1 Four‐Way Crossover TQT Study

#### 2.2.1 Study Design

This was a Phase 1 randomized, placebo‐controlled, double‐blind, single‐centre, four‐way crossover TQT study to assess the effect of single baxdrostat 16 and 32 mg oral doses (tablet formulation) on QTcF versus placebo, with moxifloxacin as a positive control, in healthy adult participants. Participants were randomized to receive a single dose of either baxdrostat 16 mg, baxdrostat 32 mg, placebo, or open‐label moxifloxacin 400 mg, in one of four treatment sequences. Baxdrostat doses were selected to allow for an estimation of the effect of the highest clinically relevant exposures of baxdrostat on the ΔΔQTcF interval. Doses were administered after an overnight fast of ≥ 10 h with 240 mL of water; participants could drink water until 1 h before dosing. There were six visits: ≤ 28 days of screening (Visit 1); four treatment periods (Visits 2–5) from Days −1 to 3 (48 h post‐dose), each separated by a washout period of 7–9 days; and a final follow‐up (Visit 6) within 7–10 days of discharge after Visit 5.

#### 2.2.2 Study Participants

Participants were included if they were aged 18–55 years; deemed to be healthy, with suitable veins for cannulation or repeated venipuncture; not pregnant or currently lactating; of non‐child‐bearing potential or adherent to specified contraception methods; had a BMI of 19–30 kg/m^2^; and weighed ≥ 50 kg. Exclusion criteria included a history of a clinically significant disease/disorder that put the participant or the study results at risk; history of risk factors for TdP (e.g., heart failure, clinically important bradycardia, electrolyte disturbances or family history of long QT syndrome); family history of sudden cardiac death of a first‐degree relative aged < 50 years; and any clinically significant abnormalities in vital signs (systolic BP <90 or ≥ 140 mmHg; diastolic BP < 50 or ≥ 90 mmHg; HR < 50 or > 90 bpm), 12‐lead resting ECG (abnormalities in rhythm, conduction or morphology that may interfere with QTcF interpretation), clinical chemistry, hematology or urinalysis at screening and first admission.

#### 2.2.3 Endpoints

The primary endpoint was the effect of single doses of baxdrostat 16 and 32 mg on QTcF compared with placebo using a C‐QTcF analysis (to estimate ΔΔQTcF). Secondary endpoints included the effect of baxdrostat 16 and 32 mg on HR, QTcF, QT, RR, PR, and QRS intervals at defined time points; determination of the assay sensitivity of the study with a small QTc effect from 400 mg oral moxifloxacin (positive control) using a C‐QTcF analysis; PK characteristics of baxdrostat; and safety and tolerability of baxdrostat.

#### 2.2.4 Cardiodynamic Assessment

Time‐course continuous dECG data were collected for inclusion in the C‐QTc analysis. dECG files of 20 min at pre‐dose on Day 1 (baseline) and ≥ 5 min at 30, 60, and 90 min, and 2, 3, 4, 6, 8, 12, 24, and 48 h post‐dose were collected using 12‐lead Telemetry Surveyor equipment (Mortara, Wisconsin, USA). All dECG recordings were obtained once participants had rested in a supine position for ≥ 10 min and just prior to PK sampling. Lead V2 was used as the primary lead, lead V5 as the primary back‐up lead, and lead II as the secondary back‐up lead for all time points when lead V2 was found to be unsuitable for analysis or evaluation. The analysis of all dECGs was performed in a blind manner using the EClysis system Version ≥ 4.0 [26] (ECG Analysis, AstraZeneca proprietary tool, Mölndal, Sweden) at the AstraZeneca ECG Centre (AstraZeneca ECG Core Lab, Mölndal, Sweden).

dECG data were used to derive the following parameters: HR (HR=60/RR), RR, PR, QRS, QT and QTcF where the RR interval was in seconds and QT was in ms. Calculation of dECG parameters was performed after smoothing of QT and RR data on an individual participant basis by Parexel (Durham, North Carolina, USA) in a blinded manner. Full details of the equations used in the analysis are reported in the Methods S1.

A linear mixed‐effect C‐QTcF model was used for the primary analysis. The dependent variable was ΔΔQTcF using individual time‐matched placebo for a given participant, which accounted for the diurnal change in QTc time course from the cross‐over study design; baxdrostat plasma concentrations were the independent variable. Fixed effects were an intercept in the absence of a treatment effect, the slope of the assumed linear association between concentration and ΔΔQTcF, and baseline QTcF. Random effects were included on the intercept term and the slope. The random effects were assumed to be normally distributed around 0 and with an unstructured covariance matrix, as recommended in the 2018 ICH Scientific White Paper [27]. ΔΔQTcF at the concentration of interest was estimated. Based on data derived from patients with resistant hypertension receiving baxdrostat 2 mg once‐daily, the concentration of interest for hypertension indication was predicted to be 86 ng/mL. This is 2 times the geometric mean of the maximum observed plasma concentration (*C*_max_) at steady state (coefficient of variation %) = 43.1 (28) ng/mL, adjusted for additional potential intrinsic and extrinsic factors (such as effects of drug–drug interactions). As with the MAD study, the ICH E14 threshold for regulatory concern (5 ms as evidenced by an upper bound of the 95% CI around the mean effect on QTc of 10 ms) was used to exclude a prolonging effect on QTc [22, 23].

The C‐ΔΔQTc model used for the primary analysis, with moxifloxacin as the active treatment instead of baxdrostat, was used to analyze the moxifloxacin positive control data. Assay sensitivity was demonstrated if the predicted QT effect (lower bound of the two‐sided 90% CI of ΔΔQTcF) was > 5 ms at the observed geometric mean *C*_max_ from a single 400 mg moxifloxacin dose [23].

Exploratory analyses were conducted as described above for the MAD study to determine if the assumptions in the modeling analysis were met, with a linear relationship between baxdrostat concentrations and ΔΔQTcF, without hysteresis. Exploratory graphical analyses and linear mixed‐effects model development and simulation for the TQT study were performed using SAS PROC MIXED Version ≥ 9.4 (SAS, Cary, North Carolina, USA).

#### 2.2.5 Pharmacokinetic Assessment

Blood samples for PK measurements in participants in the safety population who received baxdrostat were collected pre‐dose, 30, 60, 90 min, 2, 3, 4, 6, 8, 12, 24, and 48 h post‐dose. PK variables for plasma baxdrostat were analyzed by AstraZeneca R&D (Cambridge, United Kingdom) using non‐compartmental methods with Phoenix WinNonlin Version ≥ 8.3 (Certara, Radnor, Pennsylvania, USA) and/or SAS Version ≥ 9.2. Parameters included area under the plasma concentration‐time curve from zero to infinity (AUC_inf_); area under the plasma concentration‐curve from zero to the last quantifiable concentration (AUC_last_); *C*_max_; and time to peak or maximum observed concentration or response following drug administration (*t*_max_).

#### 2.2.6 Safety and Tolerability

The safety and tolerability profile of baxdrostat was evaluated by assessment of AEs, vital signs, 12‐lead ECGs, continuous ECG telemetry, physical examination, and laboratory assessments (hematology, clinical chemistry, and urinalysis).

#### 2.2.7 Statistical Analyses

Descriptive statistics were used for plasma concentrations and PK parameters, dECG data, and safety and tolerability assessments. A sample size of 24 evaluable participants was used to provide more than 91% power (estimated using a paired t‐test) to exclude that baxdrostat causes ≥ 10 ms QTc effect.

## 3 Results

### 3.1 Baxdrostat 0.5–5.0 Mg: Phase 1 MAD Study and C‐QTc Analysis

Complete baseline characteristics, PK, PD, and safety and tolerability results from the 56 healthy participants included in the Phase 1 MAD study have been previously reported [21].

**FIGURE 1:**
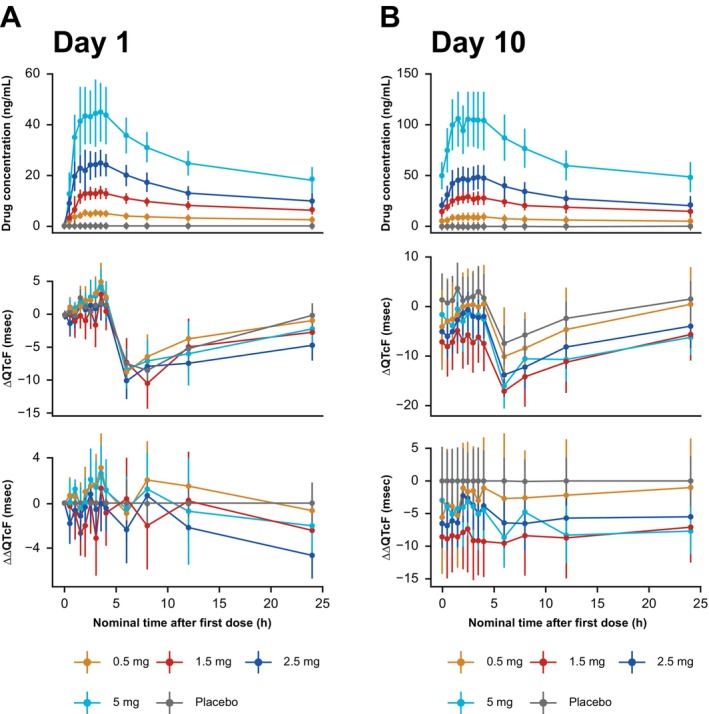
Baxdrostat plasma concentration, ΔQTcF and ΔΔQTcF over time per dose group at Day 1 (A) and Day 10 (B) (MAD study). Data points connected with lines represent mean values within one dosing group. Error bars represent 90% CI for HR measurements and SD for plasma drug concentrations. ΔQTcF, baseline‐adjusted QTcF; ΔΔQTcF, placebo‐corrected ΔQTcF; CI, confidence interval; HR, heart rate; QTcF, QT interval corrected for HR using Fridericia's formula; SD, standard deviation.

Although a potential effect of baxdrostat on HR was observed on Day 10, mean placebo‐corrected and baseline‐adjusted HR (ΔΔHR) was < 10 bpm at all timepoints and therefore QT correction for HR using Fridericia's formula was appropriate (Figure S1). Time delay between plasma baxdrostat distribution and QTcF was evaluated by comparing time courses of plasma drug concentration, mean ΔQTcF, and ΔΔQTcF (Figure 1). On Day 10, ΔΔQTcF was lower in the participants who received baxdrostat compared with placebo (Figure 1), which might potentially be due to the small increase in HR from baxdrostat on Day 10 (Figure S1).

There was no apparent difference in the ΔQTcF time course between participants with (Cohort A) or without (Cohort D) Cortrosyn challenge in both baxdrostat and placebo groups, nor did there appear to be a relationship between dietary salt restrictions and ΔQTc. As Cortrosyn challenge and dietary salt restrictions did not impact ΔQTcF, it was not necessary to correct for this in the linear model (Figure S2). The locally estimated scatterplot smoothing (LOESS) line tracked the linear regression line over the span of the observed baxdrostat concentrations, which supported the appropriateness of using a linear C‐QTcF relationship model for analysis (Figure S3).

**FIGURE 2:**
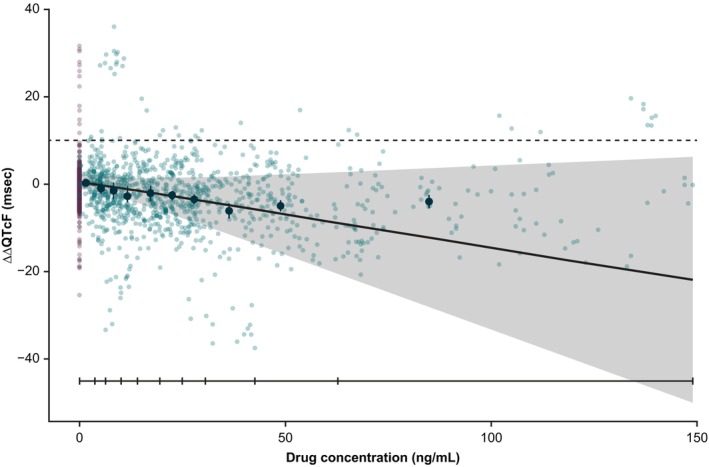
Model‐derived estimates of ΔΔQTcF against baxdrostat concentration calculated for the observed concentration range (MAD study). Black line with shaded area represents the mean model estimates with 90% CI. Solid black line with tick marks represents deciles bounds. Dashed black line represents reference line for 10 ms. Purple dots represent deciles of data with 90% CIs. ΔΔQTcF, baseline‐adjusted and placebo‐corrected QTcF; CI, confidence interval; HR, heart rate; QTcF, QT interval corrected for HR using Fridericia's formula.

The upper bound of the two‐sided 90% CI of the model‐derived mean ΔΔQTcF plotted against baxdrostat concentration was < 10 ms (consistent with ICH E14 guidance [22, 23]) for the entire observed baxdrostat concentration range (slope estimate: −0.15 [95% CI −0.38, 0.07]) (Figure 2).

### 3.2 Baxdrostat 16 and 32 mg: Phase 1 Four‐Way Crossover TQT Study

#### 3.2.1 Participants

Of the 64 participants enrolled in the study, 28 (comprising the safety and PK analysis sets) were randomized to one of four treatment sequences (Table 1), and 27 (comprising the PD analysis set) completed the study. Randomized participants were between 25 and 55 years of age, and most were male (60.7%; 17/28), white (92.9%; 26/28), and not Hispanic or Latino (100%; 28/28) (Table 1). The mean (±SD) height and weight of participants were 174.6 (±9.6) cm and 75.6 (±11.2) kg, respectively; mean (±SD) BMI was 24.7 (±2.4) kg/m^2^.

**TABLE 1:** Baseline demographics and characteristics in randomized participants (TQT study).

Parameter	Treatment				Total (*N* = 28)
	A/B/C/D (*n* = 7)	B/D/A/C (*n* = 7)	C/A/D/B (*n* = 7)	D/C/B/A (*n* = 7)	
Mean (SD) age, years	41.1 (11.1)	46.4 (6.8)	44.4 (9.6)	43.6 (9.7)	43.9 (9.1)
Sex, *n* (%)					
Male	4 (57.1)	5 (71.4)	6 (85.7)	2 (28.6)	17 (60.7)
Female	3 (42.9)	2 (28.6)	1 (14.3)	5 (71.4)	11 (39.3)
Race, *n* (%)					
White^a^	6 (85.7)	7 (100)	6 (85.7)	7 (100)	26 (92.9)
Mean (SD) height, cm	177.4 (10.8)	177.4 (8.2)	176.3 (8.3)	167.4 (9.1)	174.6 (9.6)
Mean (SD) weight, kg	78.1 (13.7)	80.1 (11.5)	77.9 (7.4)	66.4 (7.4)	75.6 (11.2)
Mean (SD) BMI, kg/m^2^	24.6 (1.8)	25.4 (3.2)	25.1 (1.9)	23.7 (2.5)	24.7 (2.4)

*Note:* Data are presented as mean (SD) or *n* (%). Treatments: (A) baxdrostat 16 mg, (B) baxdrostat 32 mg, (C) placebo and (D) moxifloxacin 400 mg.

Abbreviations: BMI, body mass index; SD, standard deviation.

^a^ There were no Hispanic or Latino participants.

#### 3.2.2 Pharmacokinetic Assessment

**FIGURE 3:**
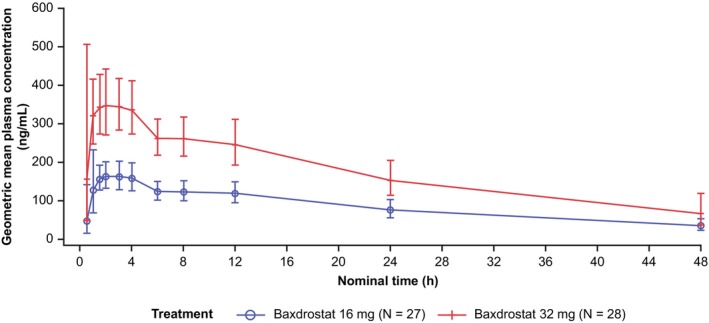
Geometric mean (± gSD) plasma concentration (ng/mL) of baxdrostat over time (linear scale) in the PK analysis set (TQT study). The PK analysis set consisted of all participants in the safety analysis set who received baxdrostat and who had evaluable PK data, with no important protocol deviations thought to impact on the analysis of the PK data. All participants received single oral dose of 16 or 32 mg of baxdrostat, and concentrations were measurable up to last time point, 48 h post‐dose. gSD, geometric standard deviation; PK, pharmacokinetic.

Pharmacokinetic parameters of baxdrostat 16 and 32 mg doses are reported in Table 4. Exposure to baxdrostat was demonstrated in all healthy participants after dosing at 16 and 32 mg. The individual maximum plasma baxdrostat concentration occurred between 0.5 and 4 h post‐dose and ranged from 117 to 280 ng/mL after the baxdrostat 16 mg dose and from 260 to 617 ng/mL after the baxdrostat 32 mg dose. Following the peak, plasma concentrations declined in a bi‐phasic manner, remaining above the limit of quantification of the assay for up to 48 h after dosing in all healthy participants (Figure 3). Overall, baxdrostat exposure was approximately linear for the 16 and 32 mg doses in terms of the geometric means of C_max_ (180.7 ng/mL vs. 412.1 ng/mL, respectively) and AUC_inf_ (5265 h × ng/mL vs. 10 630 h × ng/mL, respectively) (Table 4). Respective median *t*_max_ for 16 and 32 mg doses was 2.0 and 1.5 h.

#### 3.2.3 Cardiodynamic Assessment

**FIGURE 4:**
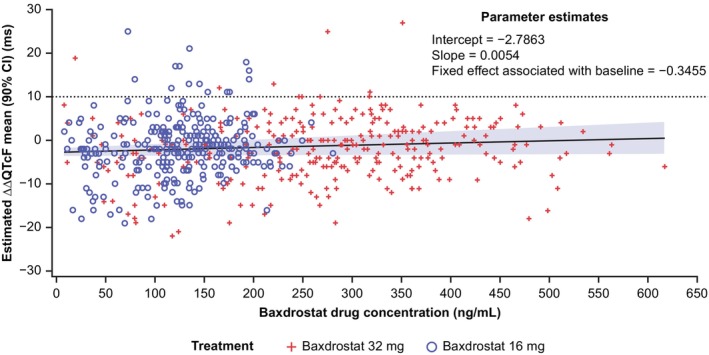
Linear mixed‐effect C‐QTcF model of ΔΔQTcF and baxdrostat concentrations in the PD analysis set (TQT study). Data shown are from 28 participants in the PD analysis set. ΔΔQTcF using individual time‐matched placebo for a given participant was the dependent variable. Fixed effect was associated with difference between baseline in the active arms (baxdrostat 16 or 32 mg) and baseline in the placebo arm. Random effects were intercept and slope associated with baxdrostat concentration. ΔΔQTcF, baseline‐corrected and placebo‐adjusted QTcF; C, concentration; CI, confidence interval; HR, heart rate; PD, pharmacodynamic; QTcF, QT interval corrected for HR using Fridericia's formula.

From the linear mixed‐effect model of ΔΔQTcF and baxdrostat (Figure 4, Figure S4), the estimated C‐QTc slope was equal to 0.0054 ms (90% CI 0.0002, 0.0106) and was of borderline statistical significance (Table 2). At the baxdrostat concentration of interest, and at the geometric mean *C*_max_ of baxdrostat 16 and 32 mg, the upper bounds of the two‐sided 90% CI for the ΔΔQTcF mean estimate were < 10 ms (Table 2). This shows that treatment with baxdrostat 16 and 32 mg did not result in a clinically relevant prolongation of cardiac repolarisation (assessed by the QTcF interval).

**TABLE 2:** Summary of ΔΔQTcF estimates and relationships with baxdrostat concentration in participants in the PD analysis set (TQT study).

Parameter	Baxdrostat (*N* = 27)
Mean ΔΔQTcF (90% CI) at pre‐specified baxdrostat concentration of interest [86 ng/mL]^a^	−2.3 (−3.7, −0.9)
Mean ΔΔQTcF (90% CI) at gmean of *C*_max_ of baxdrostat 16 mg [180.7 ng/mL]	−1.8 (−3.6, 0.0)
Mean ΔΔQTcF (90% CI) at gmean of *C*_max_ of baxdrostat 32 mg [412.1 ng/mL]	0.6 (−3.4, 2.2)
Modeled ΔΔQTcF and baxdrostat concentration relationships (95% CI)	
Intercept	−2.7863 (−4.0500, −1.5225)
Slope	0.0054 (0.0002, 0.0106)
Fixed effect associated with centred baseline QTcF	−0.3455 (−0.4400, −0.2510)

*Note:* The analysis of the relationship between plasma concentrations of baxdrostat and ΔΔQTc was performed using a linear mixed‐effect model. ΔΔQTcF using individual time‐matched placebo for a given participant was the dependent variable. Fixed effect was associated with differences between baseline in the active arms (baxdrostat 16 or 32 mg) and baseline in the placebo arm. Random effects are intercept and slope associated with baxdrostat concentration. Using the model, ΔΔQTcF was estimated at given concentrations.

Abbreviations: ΔΔQTcF, baseline‐corrected and placebo‐adjusted QTcF; CI, confidence interval; *C*_max_, observed maximum plasma concentration; gCV%, geometric coefficient of variation; gmean, geometric mean; HR, heart rate; PD, pharmacodynamic; QTcF, QT interval corrected for HR using Fridericia's formula.

^a^ The concentration of interest in the hypertension indication was predicted to be 86 ng/mL (*C*_max_ at steady state = 43.1 [25] ng/mL (gmean, [gCV%])) after baxdrostat 2 mg once daily dosing in patients with resistant hypertension and adjusted for potential intrinsic and extrinsic factors (such as drug–drug interactions) by a factor of two.

**FIGURE 5:**
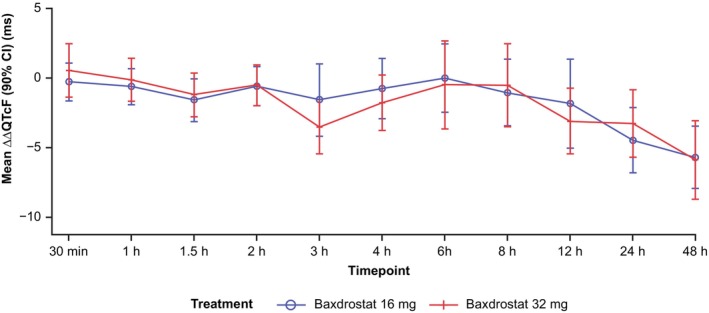
Mean ΔΔQTcF over time by baxdrostat 16 mg and 32 mg groups in the PD analysis set (TQT study). Data shown are from 28 participants in the PD analysis set. The x‐axis does not follow a linear timescale. ΔΔQTcF, baseline‐corrected and placebo adjusted QTcF; CI, confidence interval; HR, heart rate; PD, pharmacodynamic; QTcF, QT interval corrected for HR using Fridericia's formula.

Over the 48 h assessment period, mean HR, QTcF, RR, PR, and QRS intervals observed with baxdrostat at 16 and 32 mg were similar to those observed with placebo. In the baxdrostat 16 and 32 mg groups, no notable changes in ΔHR, ΔRR, ΔPR, and ΔQRS intervals over time were observed, nor were any for ΔΔHR, ΔΔRR, ΔΔPR, and ΔΔQRS intervals. No notable changes were observed in ΔΔQTcF over time and, consistent with the C‐QTcF analysis, the upper bounds of the two‐sided 90% CI of ΔΔQTcF for baxdrostat 16 and 32 mg were < 10 ms across the whole concentration range (Figure 5, Figure S5).

Assay sensitivity was investigated using a C‐QTcF analysis with oral moxifloxacin 400 mg as a positive control. The estimated C‐QTc slope was positive and equal to 0.0044 ms (with a 90% CI of 0.0031, 0.0057), and parameters of the model were statistically significant (Table 3, Figure S6). The lower bound of the two‐sided 90% CI of estimated ΔΔQTcF was 8.8 ms at the geometric mean C_max_ of moxifloxacin (Table 3); this was above the 5 ms threshold at which assay sensitivity was deemed as met, which confirmed the sensitivity of the QT assay. This was further supported by the mean ΔΔQTcF of 10.7 ms at *C*_max_. In addition, exploratory analyses confirmed modeling assumptions were met, including the validity of the correction made for heart rate (Figure S7) and the existence of a linear relationship between baxdrostat concentrations and ΔΔQTcF, without hysteresis (Figures S8 and S9).

**TABLE 3:** Summary of ΔΔQTcF estimates and assay sensitivity modeling with moxifloxacin 400 mg in participants in the PD analysis set (TQT study).

Parameter	Moxifloxacin 400 mg (*N* = 27)
Estimates (95% CI) from assay sensitivity modeling	
Intercept	2.5997 (0.7060, 4.4934)
Slope	0.0044 (0.0031, 0.0057)
Fixed effect associated with baseline QTcF	−0.5017 (−0.6597, −0.3437)
Estimate (90% CI) ΔΔQTcF at gmean of C_max_ of moxifloxacin [2367.1 ng/mL]	13.0 (8.8, 17.2)

*Note:* The analysis of the relationship between plasma concentrations of moxifloxacin and ΔΔQTc was performed using a linear mixed‐effect model. ΔΔQTcF using individual time‐matched placebo for a given participant was the dependent variable. Fixed effect was associated with differences between baseline in the active arm and baseline in the placebo arm. Random effects are intercept and slope associated with moxifloxacin concentration.

Abbreviations: ΔΔQTcF, baseline‐corrected and placebo adjusted QTcF; CI, confidence interval; *C*_max_, observed maximum plasma concentration; gmean, geometric mean; HR, heart rate; PD, pharmacodynamic; QTcF, QT interval corrected for HR using Fridericia's formula.

**TABLE 4:** Summary of PK parameters of baxdrostat in participants in the PK analysis set (TQT study).

Parameter	Baxdrostat 16 mg (*N* = 27)	Baxdrostat 32 mg (*N* = 28)
*C*_max_ (ng/mL)	180.7 (20.5)	412.1 (22.7)
AUC_last_ (h*ng/mL)	4052.0 (24.1)	8350.0 (22.8)
AUC_inf_ (h*ng/mL)	5265.0 (30.9)	10630.0 (29.7)
*t*_max_ (h)	2.0 (0.5–4.0)	1.5 (0.5–4.0)

*Note:* Data are shown as gmean (gCV%) for all except median (range) for *t*_max_.

Abbreviations: AUC_inf_, area under plasma concentration time curve from zero to infinity; AUC_last_, area under the plasma concentration curve from zero to the last quantifiable concentration; *C*_max_, observed maximum plasma concentration; gCV%, geometric coefficient of variation; gmean, geometric mean; PK, pharmacokinetic; *t*_max_, time to reach maximum plasma concentration.

**TABLE 5:** Overall summary of adverse events in participants in the safety analysis set (TQT study).

Adverse event, *n* (%)	Baxdrostat 16 mg (*N* = 27)	Baxdrostat 32 mg (*N* = 28)	Placebo (*N* = 27)	Moxifloxacin 400 mg (*N* = 28)
Any AE	3 (11.1)	4 (14.3)	4 (14.8)	9 (32.1)
Any SAE	0	0	0	0
Any SAE with outcome death	0	0	0	0
Any AE leading to discontinuation of study drug	0	0	0	0
Any possibly drug‐related^a^ AE	1 (3.7)	2 (7.1)	0	4 (14.3)
Any possibly drug‐related^a^ SAE	0	0	0	0

*Note:* AEs with an onset date on or after the date of first dose of the study drug, up to and including the date of the final follow‐up visit for the last treatment period. AEs were assigned to the most recent treatment received prior to the onset of the AE. Participants with multiple occurrences in the same category were counted once per category regardless of the number of occurrences.

Abbreviations: AE, adverse event; SAE, serious AE.

^a^ Defined as a reasonable possibility that the AE was caused by the study drug, as assessed by the investigator.

#### 3.2.4 Safety and Tolerability

Rates of AEs reported across treatment groups were 11.1% (3/27) with baxdrostat 16 mg, 14.3% (4/28) with baxdrostat 32 mg, 14.8% (4/27) with placebo, and 32.1% (9/28) with moxifloxacin 400 mg (Table 5). No SAEs, deaths, AEs that led to the discontinuation of baxdrostat, or AEs of special interest (AESIs) were observed during the study. There were three participants with possibly related AEs (all headaches) in the baxdrostat dose group (1/27 in the 16 mg group and 2/28 in the 32 mg group), which were resolved at the end of the relevant treatment period.

No clinically significant abnormal ECG results, treatment‐emergent morphology abnormalities in dECG wave forms over time, or any AEs relating to ECGs were observed for any participant. In the baxdrostat 16 and 32 mg groups, no clinically significant abnormal telemetry results were observed. One participant in the moxifloxacin 400 mg group had an episode of non‐sustained ventricular tachycardia, which was asymptomatic; this was recorded as a moderate AE and was resolved/recovered at the end of the respective treatment period.

No clinically relevant changes or differences were observed for vital signs (systolic and diastolic BP and pulse rates) over time in the baxdrostat 16 and 32 mg groups. However, treatment‐emergent vital sign abnormalities were observed for all dose groups, including placebo. Abnormalities with an incidence of ≥ 10% of the participants were diastolic BP decrease (10.7% [3/28] with baxdrostat 32 mg; 11.1% [3/27] with placebo; 21.4% [6/28] with moxifloxacin 400 mg), diastolic BP increase (21.4% [6/28] with baxdrostat 32 mg), systolic BP decrease (10.7% [3/28] with moxifloxacin 400 mg), and pulse rate increase (14.3% [4/28] with moxifloxacin 400 mg).

## 4 Discussion

This analysis of matched PK and dECG data from two separate randomized, placebo‐controlled, phase 1 studies demonstrates that treatment with once‐daily oral baxdrostat showed no evidence of QT‐interval prolongation at clinically relevant and supratherapeutic exposures. Results were consistent between the two C–QTc modeling analyses and baxdrostat treatment did not produce QT‐interval prolongation, both at concentrations of interest and geometric mean *C*_max_ of the highest dose assessed. The maximum mean prolongation in ΔΔQTcF was ≤ 5 ms and upper bounds of all two‐sided 90% CIs were predicted to be < 10 ms across the whole concentration range observed in the MAD and TQT studies. Therefore, according to the ICH E14 guidelines, it can be concluded that baxdrostat has no QT effects of concern at clinically relevant exposures (i.e., 0.5–5.0 mg [MAD study] and 16 and 32 mg [TQT study]) [22, 23].

A prespecified linear mixed‐effects model of the relationship between plasma baxdrostat concentration and ΔQTcF adequately described the observed data and met the published criteria for quality and robustness, according to the goodness‐of‐fit plots in both studies [27]. Exploratory analyses in the MAD study indicated that all four key model assumptions were met (i.e., no effect on HR; QTcF appropriate method; no hysteresis; linear relationship). Although there was no apparent effect of baxdrostat on PR and QRS, a potential effect on HR was observed on Day 10 in the MAD study. However, as mean ΔΔHR was < 10 bpm at all time points, it was deemed that the impact of baxdrostat on HR is not significant and that QTcF is appropriate to use as a correction method for further assessments of the effects of baxdrostat on QT‐interval prolongation. Similarly, as Cortrosyn challenge and dietary salt restrictions did not impact ΔQTcF, it was deemed not necessary to correct for this in the model. Additionally, moxifloxacin was used as a positive control to assess assay sensitivity in the TQT study. At the geometric mean *C*_max_ of moxifloxacin, the lower bound of the two‐sided 90% CI of estimated ΔΔQTcF was 8.8 ms (i.e., > 5 ms threshold), confirming the sensitivity of the QT assay. In summary, we consider that these methodologies were appropriate to assess the effects of baxdrostat on QT‐interval prolongation per ICH E14 guidelines.

At the time of manuscript preparation, baxdrostat is being assessed at once‐daily oral doses of 1–2 mg for the treatment of uHTN and rHTN [15, 16, 17]. The TQT study was conducted to assess higher exposures than those covered in the MAD study, enabling support for indications requiring higher therapeutic doses. Here, we demonstrate that at the geometric mean *C*_max_ of 0.5–5, 16, and 32 mg baxdrostat, the upper bounds of the two‐sided 90% CI for the ΔΔQTcF mean estimate were < 10 ms across the whole concentration range. These data indicate that, even at supratherapeutic dose levels of 16 and 32 mg, baxdrostat did not result in a clinically relevant prolongation of cardiac repolarisation assessed by the QTcF interval. These data are consistent with previous analyses from the baxdrostat clinical development programme [21, 25] and other analyses wherein doses of 10 mg baxdrostat co‐administered with metformin also did not produce QT‐interval prolongation in healthy adults [24]. Additionally, for the 16 and 32 mg baxdrostat dose groups, there were no notable changes for ΔΔHR, ΔΔRR, ΔΔPR, and ΔΔQRS or ΔHR, ΔQTcF, ΔRR, ΔPR, and ΔQRS over time, nor were there outliers recorded on the QTcF.

After oral administration, baxdrostat is rapidly absorbed with peak concentrations observed within 4 h after dosing [21]. Baxdrostat concentrations have been observed to decline from peak in an apparent biphasic manner with a long mean half‐life of ~26–31 h [21]. Despite the supratherapeutic doses used in the TQT study, PK parameters were within the range of those observed in other baxdrostat studies [14, 24, 25], and the MAD study [21].

An exception to this was the terminal half‐life, which was slightly shorter than previously observed. A possible explanation for this observation is that final plasma samples were collected too close to the elimination half‐life. However, determination of terminal plasma half‐life was not the objective of this analysis. Exposures resulting from both the 16 and 32 mg dose levels were approximately linear, and the inter‐individual variability in the PK parameters for baxdrostat was low (i.e., < 30%).

There were no SAEs, deaths, AEs leading to discontinuation, or AESIs recorded during either study [21]. Three participants in the TQT study experienced possibly drug‐related AEs in the overall baxdrostat dose group: one participant in the 16 mg dose group and two participants in the 32 mg dose group. All the possibly drug‐related AEs were reported as headaches and were resolved at the end of the relevant treatment period. There were no clinically relevant changes for laboratory results, no treatment‐emergent hematology or chemistry abnormalities recorded, or any AEs recorded for the laboratory values during this study. While hypotension is a potential risk of baxdrostat treatment, there were no clinically relevant changes or differences observed for vital signs over time for baxdrostat dose groups (BP [systolic and diastolic] and pulse rate). There were some treatment‐emergent vital sign abnormalities in the baxdrostat 32 mg dose group; however, these abnormalities were not recorded as AEs. There were also no clinically significant abnormal telemetry or ECG results for any participants in the baxdrostat dose groups. Furthermore, there were no treatment‐emergent morphology abnormalities in the dECG wave forms over time. Overall, baxdrostat 16 and 32 mg were well tolerated in the TQT study population.

Strengths of the TQT study include the use of C‐QTc modeling as the primary analysis. This approach is in line with guidance from ICH [23] and allows for a reduced number of participants compared with historical TQT studies [27]. Further, the crossover study design required fewer participants compared with a parallel‐group approach, and because each participant acted as their own control, inter‐individual variability was eliminated. In addition, data consistency was demonstrated across both MAD and TQT studies. The maximum mean prolongation in ΔΔQTcF was ≤ 5 ms with upper bounds of all two‐sided 90% CIs < 10 ms across the whole concentration range up to baxdrostat 32 mg, noting that the highest currently assessed therapeutic dose for patients with hypertension is 2 mg. Overall, the data described in this analysis demonstrate that baxdrostat is not associated with the risk of QT‐interval prolongation at expected therapeutic concentrations.

## 5 Conclusions

The effect of baxdrostat on QTcF was below the ICH E14 threshold for regulatory concern (10 ms) in two separate studies and for doses up to 32 mg. In these studies, there was no evidence of clinically relevant QT‐interval prolongation with baxdrostat treatment, as assessed by the QTcF interval and C‐QTc modeling.

## Author Contributions

**Mikael Sunnåker:** conceptualization, data curation, formal analysis, investigation, methodology, writing – original draft, writing – review and editing. **Christian Källgren:** formal analysis, writing – original draft, writing – review and editing. **Joanna Parkinson:** conceptualization, data curation, investigation, methodology, writing – original draft, writing – review and editing. **Corina Dota:** conceptualization, data curation, investigation, methodology, writing – original draft, writing – review and editing. **Christer Gottfridsson:** conceptualization, methodology, writing – original draft, writing – review and editing. **David Janzén:** formal analysis, writing – original draft, writing – review and editing. **Anita Andersson:** conceptualization, data curation, investigation, methodology, writing – original draft, writing – review and editing. **Glenn Carlson:** conceptualization, methodology, writing – original draft, writing – review and editing.

## Ethics Statement

Both studies were performed in accordance with the principles of the Declaration of Helsinki and the International Council for Harmonization Good Clinical Practice Guidelines and were registered on ClinicalTrials.gov (NCT05500820 and NCT06194032, respectively) [29, 30]. The Phase 1 MAD and TQT studies were performed according to the AstraZeneca policy on Bioethics and Human Biological Samples.

All participants provided written informed consent before starting the studies.

## Conflicts of Interest

M.S., C.K., J.P., C.D., C.G., D.J., and G.C. are employees of AstraZeneca and own AstraZeneca stock or stock options. A.A. was an employee of AstraZeneca when the study was conducted and retains AstraZeneca stock or stock options. She is now an employee of RegSmart Life Science.

## Supporting information


**Data S1:** prp270181‐sup‐0001‐FigureS1‐S9.docx.

## Data Availability

Data underlying the findings described in this manuscript may be obtained in accordance with AstraZeneca's data sharing policy described at https://www.astrazenecaclinicaltrials.com/our‐transparency‐commitments/. Data for studies directly listed on Vivli can be requested through Vivli at www.vivli.org. Data for studies not listed on Vivli could be requested through Vivli at https://vivli.org/members/enquiries‐about‐studies‐not‐listed‐on‐the‐vivli‐platform/. AstraZeneca Vivli member page is also available outlining further details: https://vivli.org/ourmember/astrazeneca/.
