# Supplementary material for: No Evidence of QTc Interval Prolongation With Baxdrostat Treatment: Concentration–QTc Modeling Assessment
Source: Pharmacol Res Perspect. 2025 Oct 13;13(5):e70181. doi: 10.1002/prp2.70181 (PMC12518165; doi:10.1002/prp2.70181)
Supplement: Supplementary file 1 — Data S1: prp270181‐sup‐0001‐FigureS1‐S9.docx. [file PRP2-13-e70181-s001.docx]

**Supplementary Materials**

**Supplementary Methods**

***Baxdrostat 0.5–5 mg: Phase 1 MAD study and C-QTc analysis***

*Study design*

Inclusion criteria included: (1) aged 18–55 years; (2) in good health based on medical and psychiatric history, physical examination, electrocardiogram (ECG), orthostatic vital signs and routine laboratory tests (blood chemistry, haematology, coagulation and urinalysis); (3) nonsmokers; (4) body mass index (BMI) between ≥18 and ≤30 kg/m^2^; (5) demonstrate an appropriate response to cortisol stimulation (Cohorts 1 and 2) or a normal cortisol level during the inpatient run-in period (Cohorts 3–5).

Exclusion criteria included: (1) personal or family history of arrhythmias (long QT syndrome, TdP, other complex ventricular arrhythmias); (2) current or history of ventricular tachycardia and ventricular or atrial fibrillation; and (3) prolonged QT interval corrected for heart rate (HR) using Fridericia’s formula^1^ (QTcF; >450 ms).

*Low salt diet*

Participants in Cohort A consumed a diet containing 50 to 60 mEq Na^+^/day and 70 to 100 mEq K^+^/day from Day –5 until Day –1. Based on decreases in Na^+^ levels observed in some Cohort A participants during the run-in period prior to any participants receiving study drug, the diet was modified to 65 to 70 mEq Na^+^/day and 70 to 100 mEq K^+^/day from Day 1 through the remainder of the study. Participants in Cohort B consumed a low salt diet (up to 65 to 70 mEq Na^+^/day and 70 to 100 mEq K^+^/day) for the full run-in and treatment periods.

*Pharmacokinetics and electrocardiography*

Blood samples were collected to evaluate PK. On Day 1 and Day 10, blood samples were collected prior to dosing and at 0.5, 1, 1.5, 2, 2.5, 3, 3.5, 4, 6, 8 and 12 h post-dose with an additional collection to measure plasma baxdrostat concentration at 24 h post-dose prior to dosing on Day 2. On Day 10, additional blood samples were collected at 24, 36, 48, 72, 96 and 12 h post-dose. Plasma baxdrostat concentrations were analysed using a validated liquid chromatography–mass spectrometry method (LC-MS/MS) by Medpace Reference Laboratories (Cincinnati, Ohio, USA), Medpace Bioanalytical Laboratories (Cincinnati, Ohio, USA), Mercy Health—West Hospital Laboratory (Cincinnati, Ohio, USA) or Laboratory Corporation of America (Dublin, Ohio, USA).

Twelve-lead continuous dECG data collected were used in the C-QTc analysis. dECGs were extracted at −45, −30 and −15 min (to establish a robust baseline) and 0.5, 1, 1.5, 2, 2.5, 3, 3.5, 4, 6, 8, 12 and 24 h post-dose on Day 1 and Day 10. All dECG recordings were obtained once participants had rested in a supine position for ≥10 min and prior to PK sampling (except for the 6-hour time point on relevant days, when the ECG was to be taken last). A central ECG laboratory analysed the dECG data (Clario, Philadelphia, Pennsylvania, USA).

*Exploratory analyses*

For the exploratory graphical analyses, participants in the baxdrostat 2.5 mg normal- and low-salt cohorts (Cohorts A and D) were pooled into a 2.5 mg group; placebo participants in the normal- (without Cortrosyn^®^) and low- (with Cortrosyn^®^) salt cohorts (Cohorts A–E) were pooled into a placebo group.

The exploratory analyses and development/simulation of the pre-specified linear mixed effects model were performed using R software Version ≥3.5.1 and lme4 Version ≥1.1-33.

*C-QTc analysis‍*

A pre-specified linear mixed effects model (**Equation S1**) was used in the primary analysis. ΔQTcF was used as a dependent variable; observed (time-matched) baxdrostat concentration was used as an independent variable.

**Equation S1:** ${\Delta QTcF}_{ijl}=\left( \theta_{0}+h_{0,i} \right)+ \theta_{1} {TRT}_{j}+ \left( \theta_{2}+h_{2,i} \right)C_{ijl}+ \theta_{3}{TIME}_{l}+\theta_{4} \left( {QTcF}_{ijl=0} - \overline{QT{cF}_{0}} \right)+ \theta_{5}{DAY}_{10}$

In **Equation S1**, ${\Delta QTcF}_{ijl}$ is the change from baseline in $QTcF$ for participant $i$ in treatment $j$ at (nominal) time $l$; $\theta_{0}$ is the population mean intercept in the absence of a treatment effect; $h_{0,i}$ is the random effect associated with the intercept term $\theta_{0}$; $\theta_{1}$ is the fixed effect associated with treatment $j$ (0 = placebo, 1 = active drug); $\theta_{2}$ is the population mean slope of the assumed linear association between baxdrostat concentration and ${\Delta QTcF}_{ijl}$; $h_{2,i}$ is the random effect associated with the slope $\theta_{2}$; $C_{ijl}$ is the baxdrostat concentration for participant $i$ in treatment $j$ and time $l$; $\theta_{3}$ is the fixed effect associated with time; and $\theta_{4}$ is the fixed effect associated with baseline ${QTcF}_{ijl=0}$; $\overline{{QTcF}_{0}}$ is overall mean of ${QTcF}_{ijl0}$; $\theta_{5}$ is the adjustment in intercept for Day 10 compared to Day 1 (${DAY}_{10}$ = 1 on Day 10 and ${DAY}_{10}$ = 0 otherwise). Random effects were assumed to be normally distributed.

***Baxdrostat 16 mg and 32 mg: Phase 1 four-way crossover TQT study***

*Cardiodynamic assessment*

Baseline-adjusted, Fridericia heart rate (HR)-corrected QT interval (ΔQTcF) was used as the dependent variable for concentration (C)-QT modelling (**Equation S2**).

**Equation S2:** ${\Delta QTcF}_{ti}={QTcF}_{ti}-{QTcF}_{base,i}$

In **Equation S2**, ${\Delta QTcF}_{ti}$ is the change in $QTcF$ from baseline at a given timepoint ($t$) for a given participant; ${QTcF}_{ti}$ is the $QTcF$ measurement at a given timepoint for a given participant; and ${QTcF}_{base,i}$ is the baseline $QTcF$ for the participant.

Placebo-corrected ΔQTcF (ΔΔQTcF) was used as the dependent variable for C-QTcF modelling (**Equation S3**).

**Equation S3:** ${\Delta\Delta QTcF}_{ti}={\Delta QTcF}_{trt,ti}-{\Delta QTcF}_{pic,ti}$

For the C-QTcF modelling analysis (**Equation S3**), it was assumed that QTcF was independent of HR. If baxdrostat did not have a significant effect on HR (baseline-adjusted, placebo-corrected HR [ΔΔHR] <10 bpm), it was deemed that the Fridericia correction provided sufficient correction of the QT interval. Otherwise, the uncorrected QT interval was used as the dependent variable for the modelling.^2^

A linear mixed effects C-ΔΔQTc model was used for the primary analysis (**Equation S4**), under the assumption of no hysteresis and linear ΔΔQTcF and baxdrostat concentration relationship.

**Equation S4:** ${\Delta\Delta QTcF}_{i}=\left( \theta_{0}+h_{0,i} \right)+\left( \theta_{1}+h_{1,i} \right)C_{i}+ \theta_{2} ({b\Delta QTcF}_{i} - \overline{b\Delta QTcF})$

In **Equation S4**, ${\Delta\Delta QTcF}_{i}$ is the change from baseline and adjusted for time-matched placebo in $QTcF$ for participant $i$; $\theta_{0}$ is the population mean intercept; $h_{0,i}$ is the random effect associated with the intercept term $\theta_{0}$; $\theta_{1}$ is the population mean slope of the assumed linear association between baxdrostat concentration and ${\Delta\Delta QTcF}_{i}$; $h_{1,i}$ is the random effect associated with the slope $\theta_{1}$; and $C_{i}$ is the baxdrostat concentration for participant $i$ and $\theta_{2}$ is the fixed effect associated with baseline. ${b\Delta QTcF}_{i}$ is the difference between baseline in the active arms (baxdrostat 16 or 32 mg) and the baseline for placebo treatment for the participant $i$ (**Equation S5**). $\overline{b\Delta QTcF}$ is the overall mean of all individual ${b\Delta QTcF}_{i}$.

**Equation S5:** ${b\Delta QTcF}_{i}={bQTcF}_{i,trt=active}-{bQTcF}_{i,trt=placebo}$

In **Equation S5**, ${bQTcF}_{i,trt=active}$ and ${bQTcF}_{i,trt=placebo}$ correspond to baseline for active (baxdrostat 16 or 32 mg) and placebo treatment, respectively, for the participant $i$.

**Programming code in SAS used for model parameter estimation**

“proc mixed data=final;

class SUBJECT_ID;

model DDQTCF = PKCONC CENTERED_BASEQTCF/ residual solution cl alpha=0.10;

random intercept PKCONC / subject=SUBJECT_ID;

run;”

**Supplementary Figures**

**
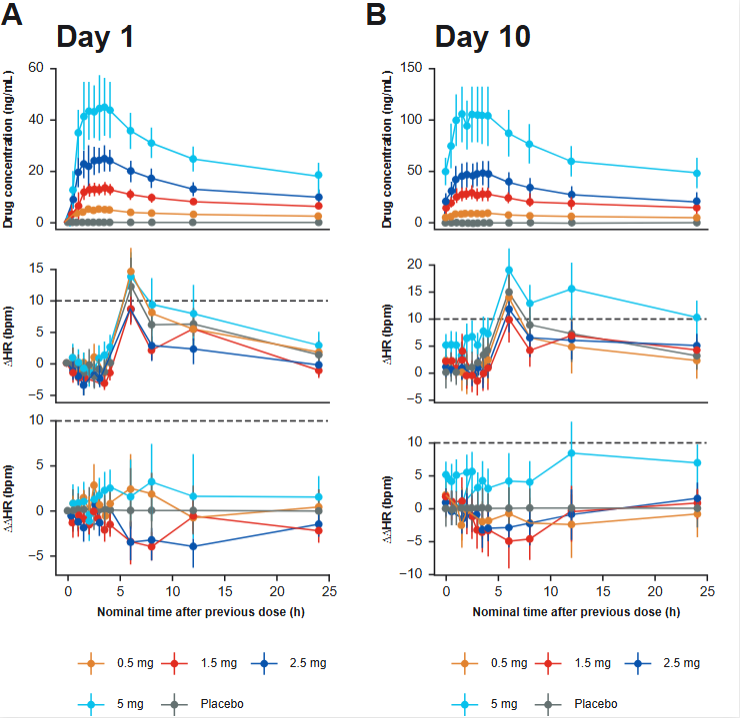
Figure S1. Evaluation of baxdrostat-induced effects on HR per dose group at Day 1 (A) and Day 10 (B) (MAD study)**

Dashed black horizontal lines represent the reference threshold at 10 bpm. Error bars represent 90% CI for HR measurements and mean ± SD for plasma drug concentration. Data points connected with lines represent mean values within one dosing group.

ΔHR, baseline-adjusted HR; ΔΔHR, placebo-corrected ΔHR; CI, confidence interval; h, hours; HR, heart rate; SD, standard deviation.

**Figure S2. Comparison of ΔQTcF in participants who received baxdrostat (2.5 mg) and pooled placebo with (low-salt diet) and without (normal-salt diet) Cortrosyn^®^ challenge on Day 1 (MAD study)**

**
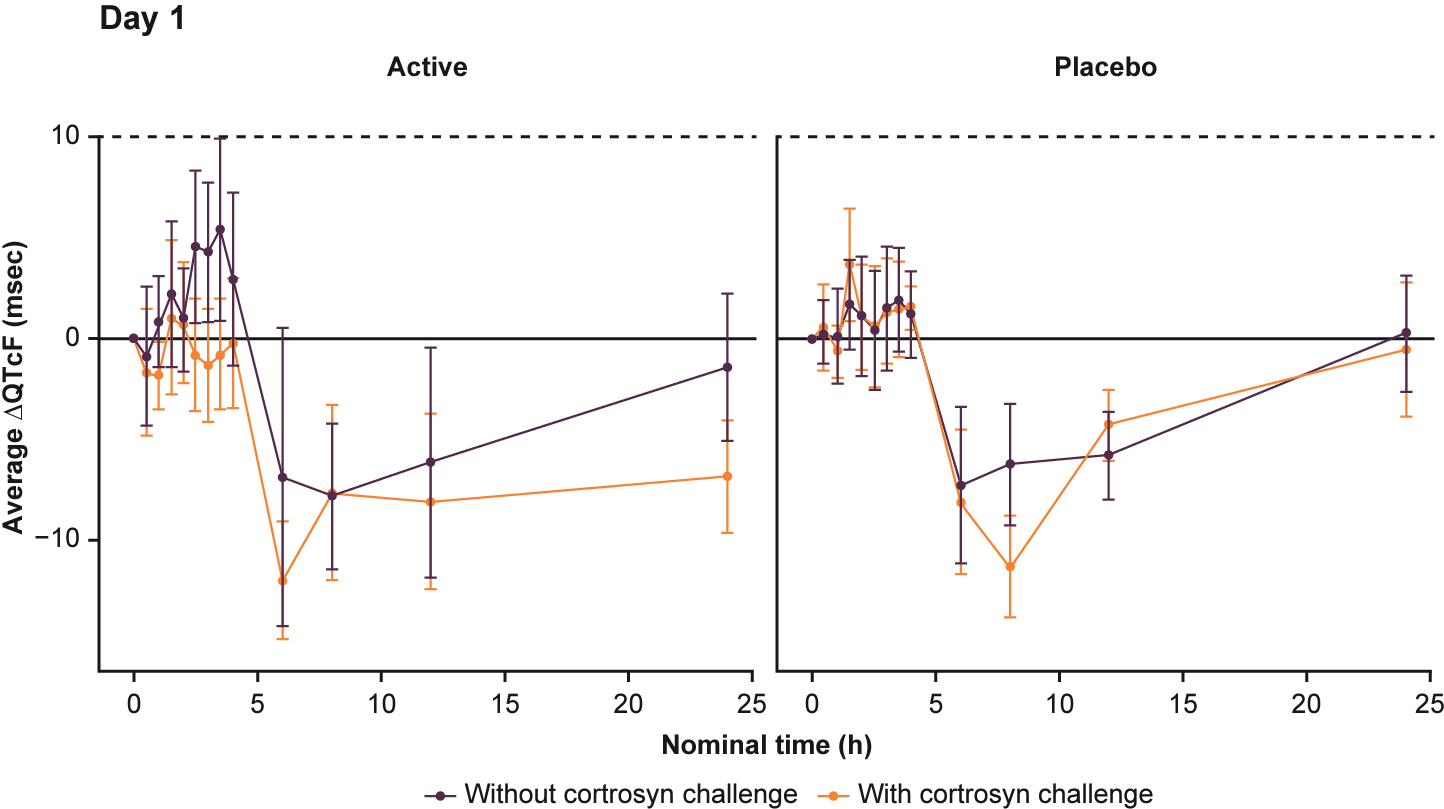
**

Participants with Cortrosyn^®^ challenge (low-salt diet cohorts) were administered Cortrosyn^®^ challenge 1h after the baxdrostat dose. Error bars represent 95% CI; lines represent the arithmetic mean values.

ΔQTcF, baseline-adjusted QTcF; CI, confidence interval; h, hour; HR, heart rate; QTcF, QT interval corrected for HR using Fridericia’s formula.


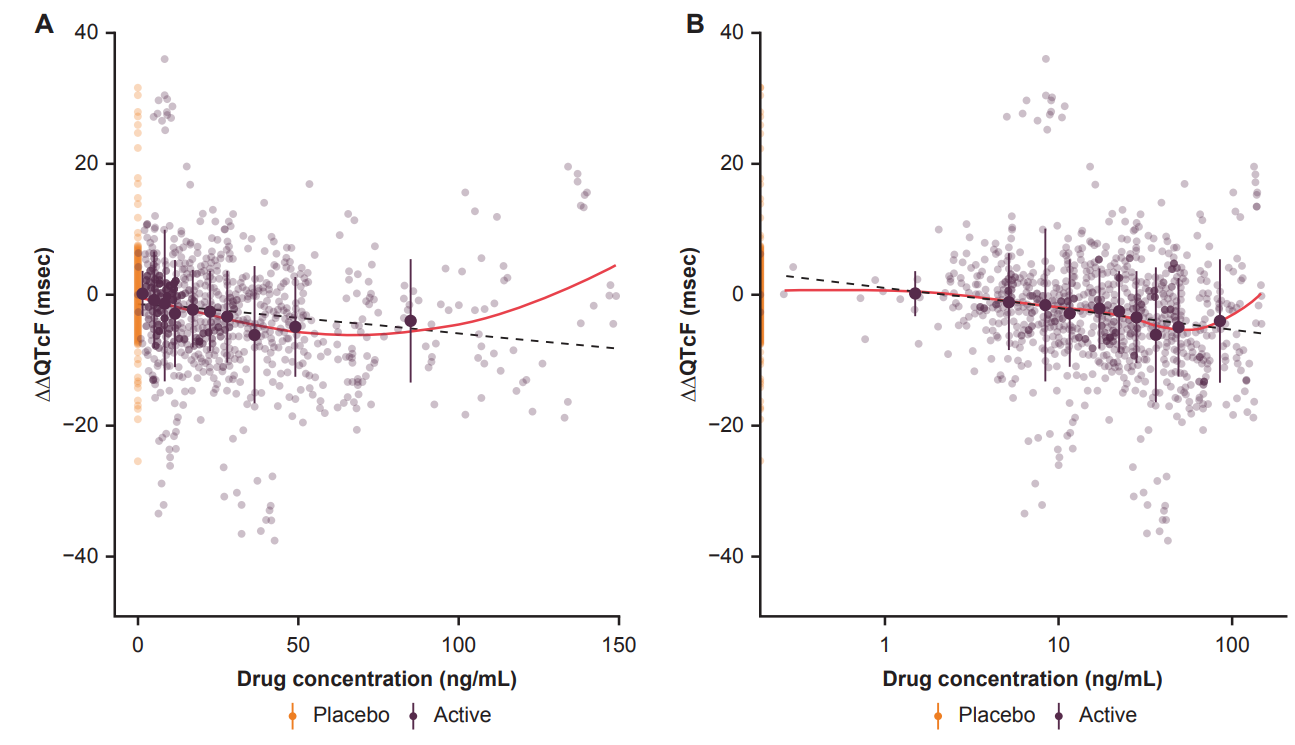
**Figure S3. Paired ΔΔQTcF and concentration data in linear (a) and logarithmic (b) scale (MAD study)**

Pale purple circles represent estimated means within deciles of the observed data; red solid lines represent LOESS; and black dashed lines represent linear regression. Error bars represent SD with respective deciles.

ΔΔQTcF, baseline-adjusted and placebo-corrected QTcF; HR, heart rate; LOESS, locally estimated scatterplot smoothing; QTcF, QT interval corrected for HR using Fridericia’s formula; SD, standard deviation.

**Figure S4. Diagnostic figures showing a summary of the modelling of ΔΔQTcF and baxdrostat concentration relationships (PD analysis set, TQT study)**

**A) Observed versus predicted values**


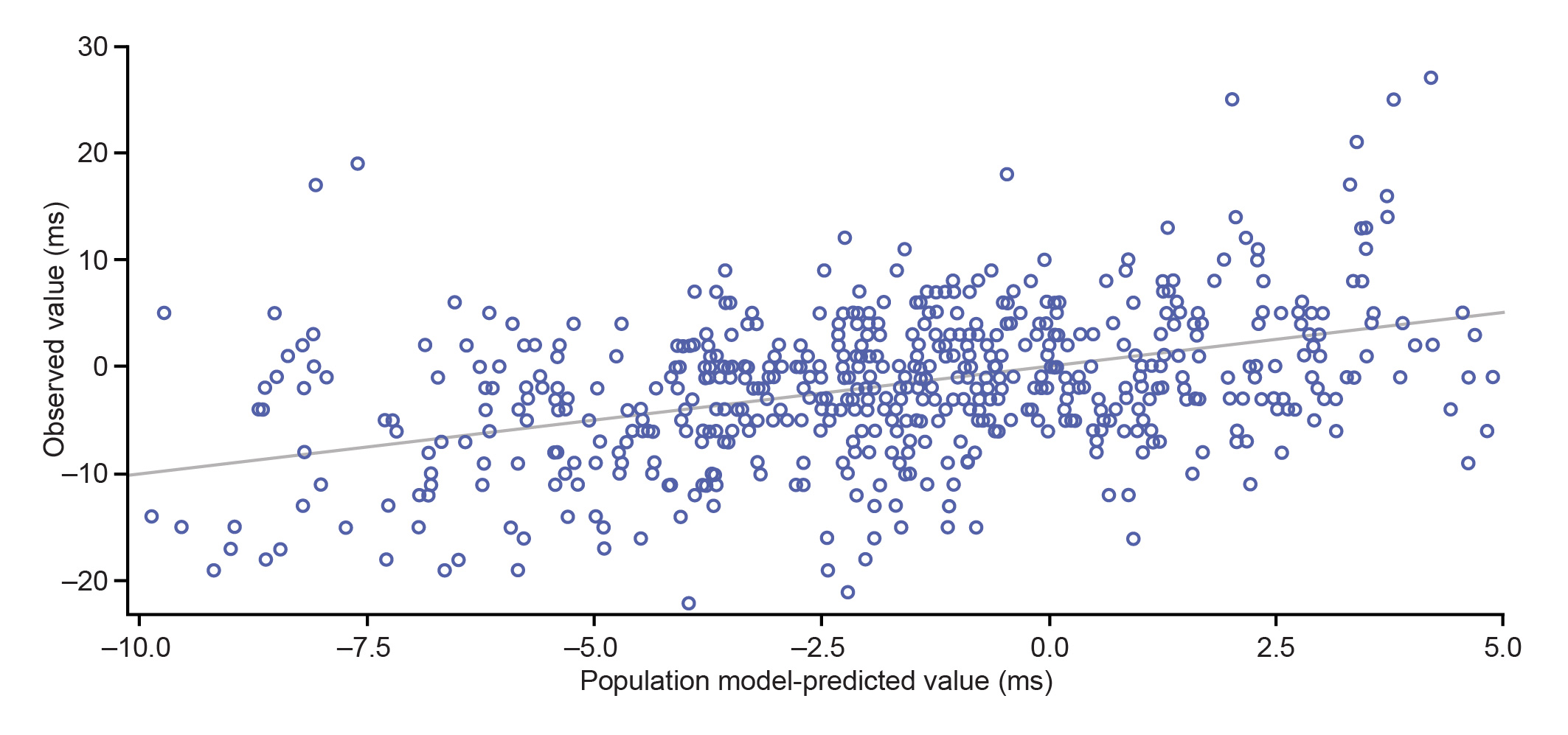


**B) Standardised residuals by concentration**

**
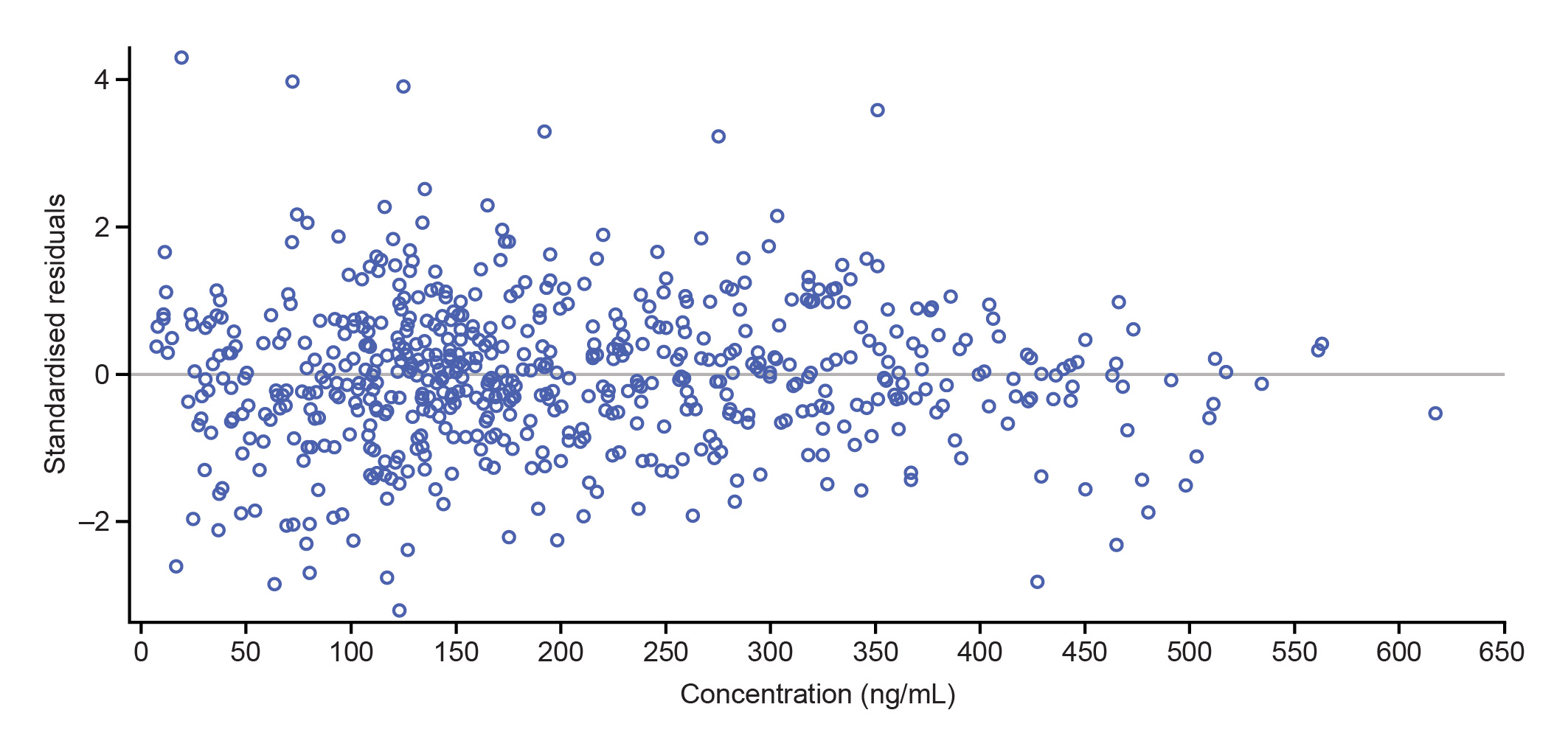
**

ΔΔQTcF, baseline-adjusted and placebo-corrected QTcF; PD, pharmacodynamic; QTcF, QT interval corrected for HR using Fridericia’s formula; TQT, thorough QT study.

**Figure S5. Digital ECG plot of mean (SD) change in ΔQTcF from baseline by treatment (PD analysis set, TQT study)**


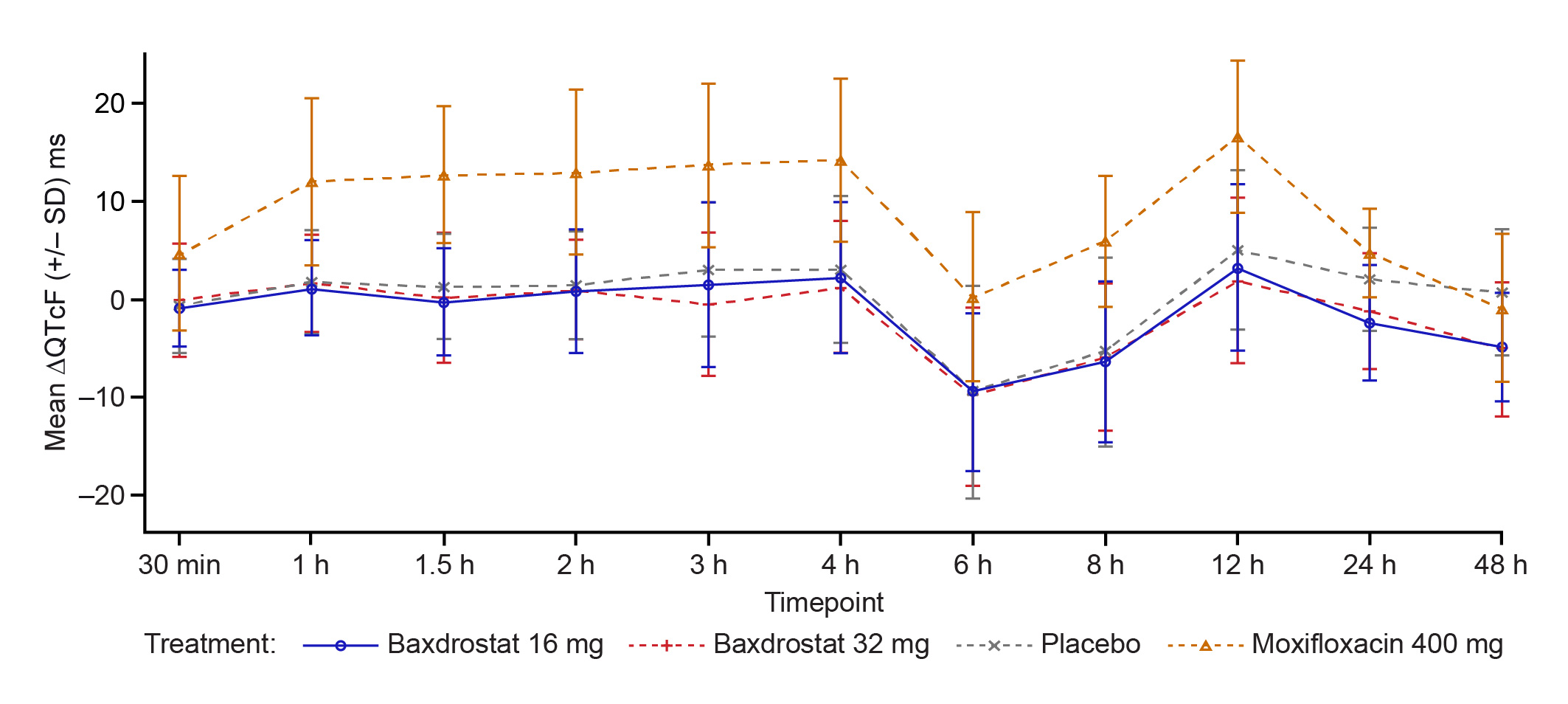


Vertical lines represent the arithmetic mean (SD).

ΔQTcF, baseline-adjusted QTcF; ECG, electrocardiogram; PD, pharmacodynamic; QTcF, QT interval corrected for HR using Fridericia’s formula; SD, standard deviation; TQT, thorough QT study.

**Figure S6. Mean ΔΔQTcF value (with 90% CI) over time for moxifloxacin (PD analysis set, TQT study)**


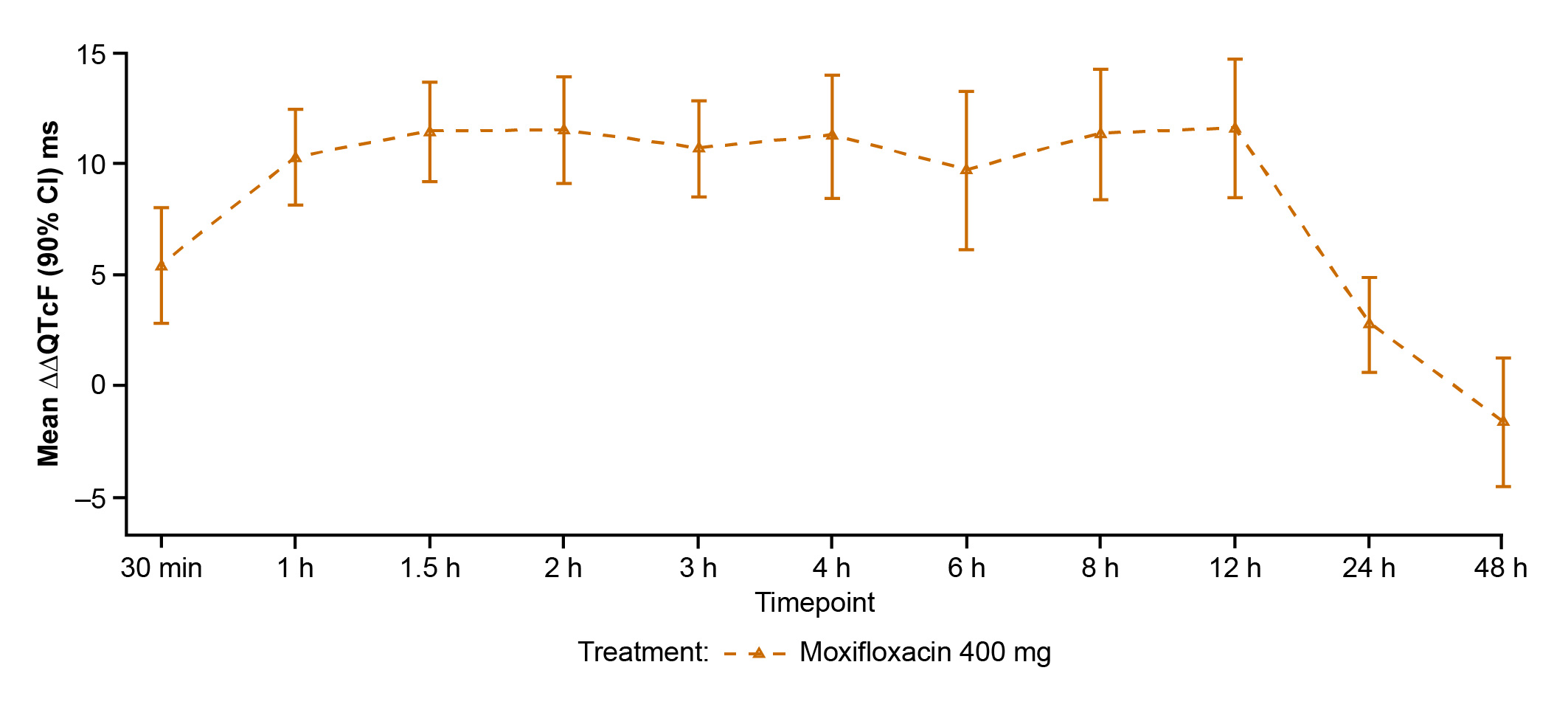


Digital ECG data are presented.

ΔΔQTcF, baseline-adjusted and placebo-corrected QTcF; CI, confidence interval; ECG, electrocardiogram; PD, pharmacodynamic; QTcF, QT interval corrected for HR using Fridericia’s formula; TQT, thorough QT study.

**Figure S7. Heart-rate correction shown by QTcF compared with RR interval for baxdrostat vs placebo (PD analysis set, TQT study)**


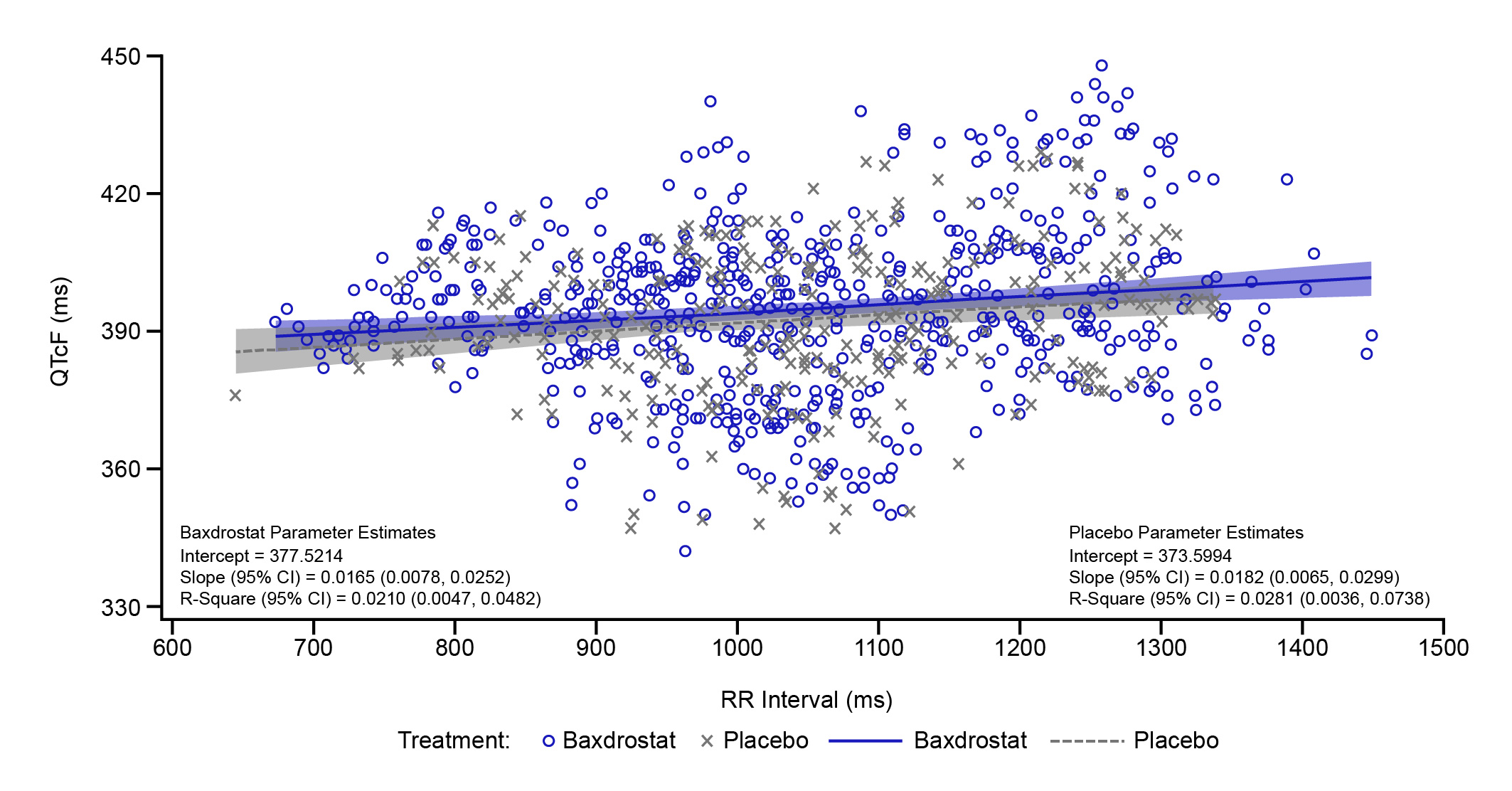


Digital ECG data are presented. Lines are linear regression lines for pooled baxdrostat and placebo with 95% CI.

CI, confidence interval; ECG, electrocardiogram; PD, pharmacodynamic; QTcF, QT interval corrected for HR using Fridericia’s formula; RR interval, time elapsed between two successive R-waves; TQT, thorough QT study.

**Figure S8. Linear scale of mean ΔΔQTcF according to various baxdrostat concentrations, PD analysis set (TQT study)**


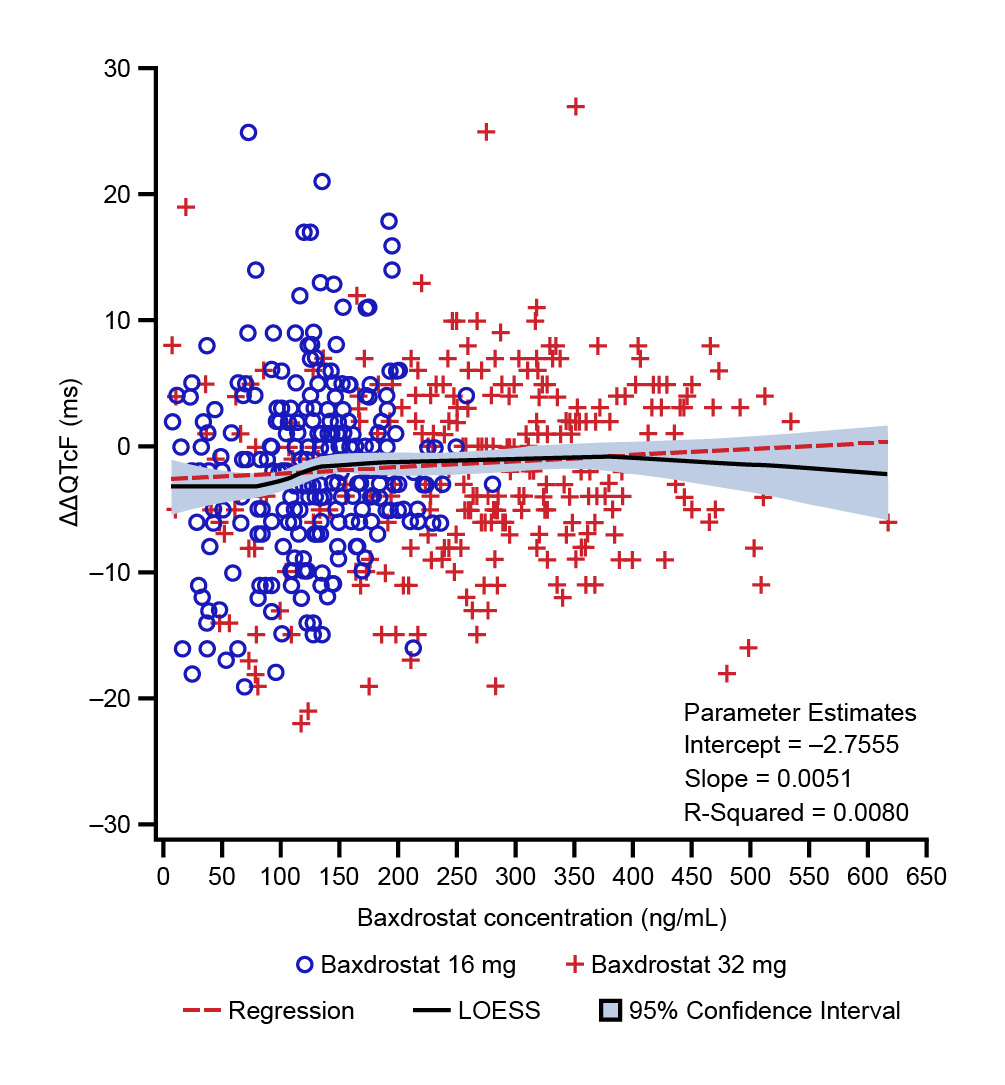


Digital ECG data are presented. Solid regression line is based on a locally estimated scatterplot smoothing line (LOESS method) with 95% CI. The dashed line represents a linear regression.

ΔΔQTcF, baseline-adjusted and placebo-corrected QTcF; CI, Confidence interval; ECG, electrocardiogram; PD, pharmacodynamic; QTcF, QT interval corrected for HR using Fridericia’s formula; TQT, thorough QT study.

**Figure S9. Hysteresis plot of mean ΔΔQTcF according to various baxdrostat concentrations, PD analysis set (TQT study)**


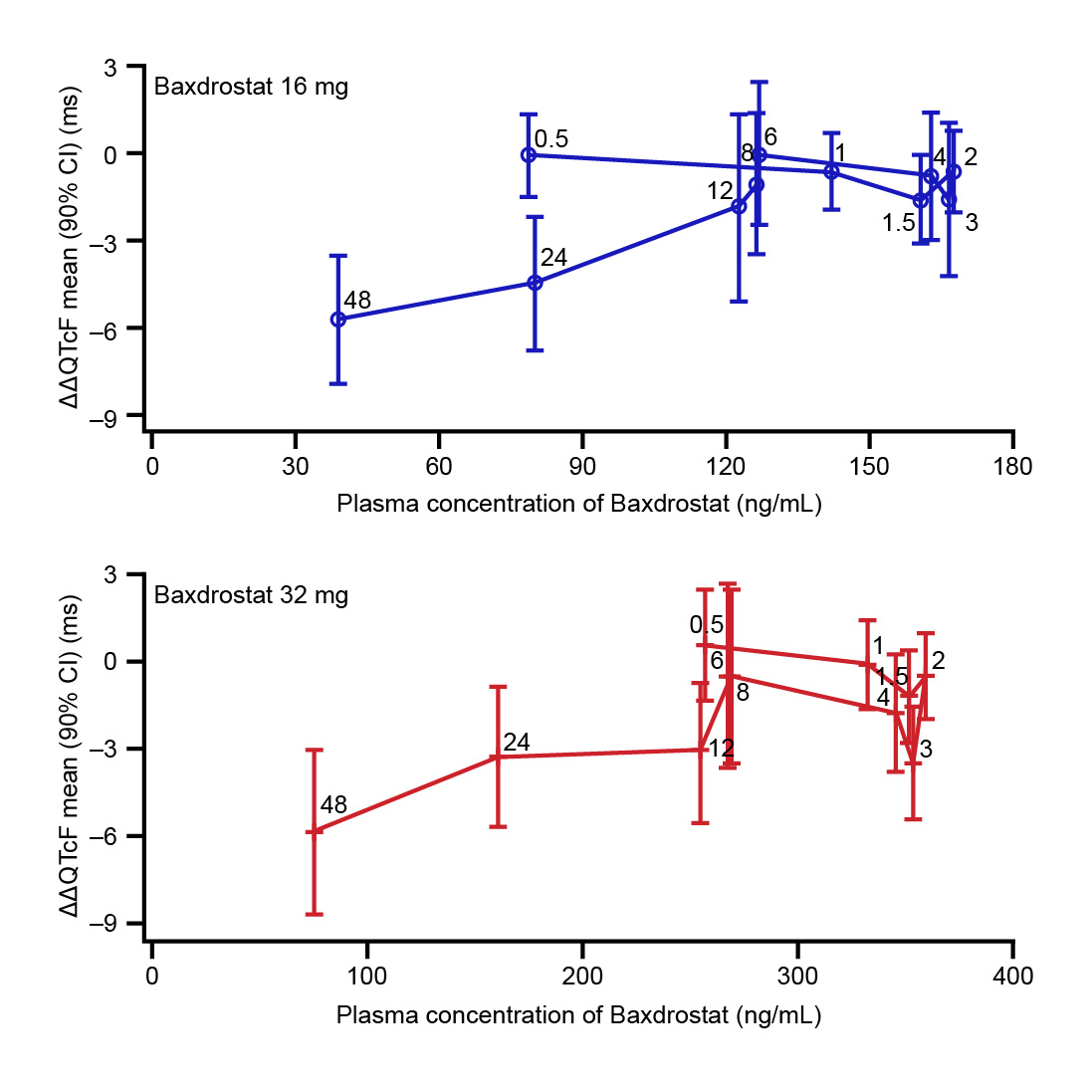


Digital ECG data are presented.

ΔΔQTcF, baseline-adjusted and placebo-corrected QTcF; CI, confidence interval; ECG, electrocardiogram; PD, pharmacodynamic; QTcF, QT interval corrected for HR using Fridericia’s formula; TQT, thorough QT study.

**Supplementary References**

1. Fridericia LS. Die Systolendauer im Elektrokardiogramm bei normalen Menschen und bei Herzkranken. Acta Medica Scandinavica. 1920;53(1):469–486. doi:https://doi.org/10.1111/j.0954-6820.1920.tb18266.x
2. Garnett CE, Zhu H, Malik M, Fossa A, Zhang J, Badilini F, et al. Methodologies to characterize the QT/corrected QT interval in the presence of drug-induced heart rate changes or other autonomic effects. *American Heart Journal*. 2012;163(6):912–30.
